# Acquired Epiblepharon Treated by Lateral Orbital and fat Decompression

**DOI:** 10.4103/0974-9233.75897

**Published:** 2011

**Authors:** Radwan Almousa, Gangadhara Sundar

**Affiliations:** 1 Department of Ophthalmology, National University Health System, 5 Lower Kent Ridge Road 119074, Singapore; 2 Corneoplastic Department, Queen Victoria Hospital, East Grinstead, West Sussex RH19 3DZ, UK

**Keywords:** Decompression, Epiblepharon, Eyelids, Orbit

## Abstract

Conventional lid surgery of acquired epiblepharon secondary to increased orbital volume can be result of under correction of the epiblepharon, because the increased orbital volume remains unaddressed. In this case report, we present a case of acquired epiblepharon, secondary to increased orbital volume, treated with orbital decompression.

## INTRODUCTION

Epiblepharon is characterized by a redundant horizontal skin-muscle fold that overrides the eyelid margin resulting in eyelash misdirection and kerato-conjunctivopathy. It can involve either the upper or lower eyelids, and is typically accompanied by an absent eyelid crease. Epiblepharon with or without kerato-conjunctival irritation may be seen either as a developmental abnormality (either as an exacerbation of the East-Asian eyelid or as an anomaly in other ethnic groups) or as an acquired abnormality. Although “developmental epiblepharon” is typically seen in children and is reported to spontaneously resolve in the first few years of life, not infrequently it persists beyond that age in East-Asians requiring surgical intervention. It should be differentiated from the entropion, which is marked by a true inward turning of the lid margin.^1^ Acquired epiblepharon is rare,^2^ and it has been described in association with obesity, thyroid related orbitopathy,^3^ systemic steroid treatment,^4^ and previous manipulation of the orbit or lower eyelid, including trauma or surgery.^3^ Spontaneous resolution was reported by control of orbital inflammation or orbital decompression for other indications.^3^

We present a case of acquired epiblepharon caused by morbid obesity treated with deep lateral wall and fat decompression with a successful outcome.

## CASE REPORT

**Figure 1:**
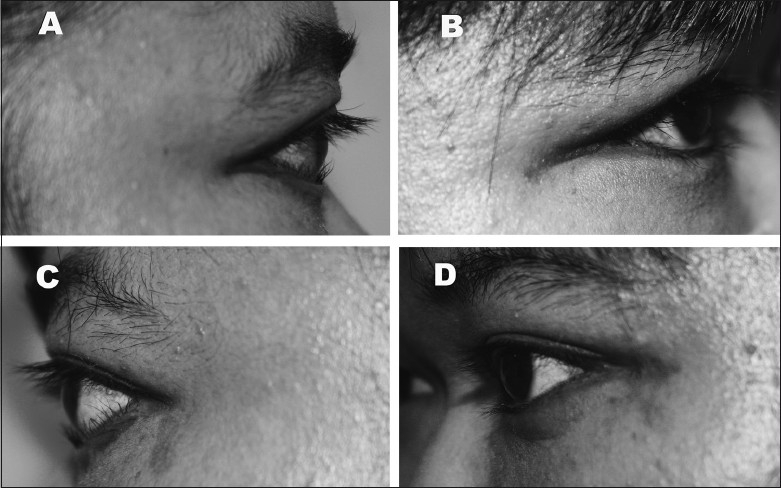
(A,C) Preoperative pictures showing the lower lid epiblepharon and the attenuated lower lid crease. (B,D) The same patient 5 weeks after the lateral wall decompression with fat debulking showing relief of epiblepharon

**Figure 2:**
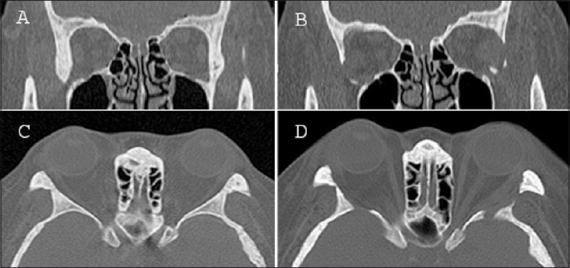
(A,B) Coronal CT scan preoperativel and 2 months postoperatively. (C,D) Axial scan for pre and postoperative orbit, showing more lateral wall decompression in the left side

A 38-year-old Malay female presented with complaints of bilateral ocular irritation of a few months duration. The patient was morbidly obese (body mass index 56.2 kg/m^2^) with a history of primary hypothyroidism and ventricular septal defect. On examination, the patient was noted to have severe bilateral lower lid epiblepharon with lash-corneal touch and corneal erosions [Figure 1] and bilateral lower eyelid retraction. Hertel exophthalmometer readings were 23 mm bilaterally, which is significant for proptosis in an East-Asian population.^5^ There was no previous documentation of proptosis and the remainder of the ophthalmic exam was normal with no evidence of orbital inflammation. An orbital computed tomography (CT) scan showed normal extraocular muscle dimensions, increased orbital fat with prolapse, and thick lateral orbital wall and marrow space with globe prolapse [Figure 2]. As the epiblepharon was presumed to have manifested from increased orbital pressure (possibly from fat volume increase of the underlying morbid obesity), the routine epiblepharon correction by skin-muscle excision (modified Hotz procedure)^6^ would have probably made her eyelid retraction worse (as is often seen in prominent eyes), and hence a minimally invasive limited orbital decompression was planned. The patient underwent lateral wall bone decompression with fat decompression. Using a high-speed neurosurgical drill, cortical bone was removed from the lacrimal gland fossa, the marrow space of the sphenoid between the superior and inferior orbital fissure, and the zygomatic marrow space on the anterior rim of the inferior orbital fissure. The intraconal fat, located between the lateral and inferior rectus muscles, was bluntly dissected out of the muscle cone and excised (2CC from the right side and 1CC from the left). Two months after surgery, following resolution of periorbital swelling and bruising, the globes were retroplaced by 7 mm and 8 mm on the right and left sides respectively and the epiblepharon resolved [Figure 1]. Postoperatively, there was a reduction of proptosis as well as resolution of epiblepharon and keratopathy.

## DISCUSSION

The proposed mechanisms of epiblepharon in East-Asians include the failure of upper or lower eyelid retractors gaining access to the overlying orbicularis/skin resulting in a redundant skin-muscle fold with lash malposition. In addition, a shallow orbit associated with prolapse of orbital fat beyond the orbital rims into the eyelid tissues, may also predispose to epiblepharon.^7^ We speculate that morbid obesity in our patient led to an increased orbital fat volume causing an override of the anterior lamella over the posterior lamella accompanied by mild lower eyelid retraction. Morbidity of orbital decompression has been significantly reduced with the advent of lateral wall and fat decompression especially in patients without preexisting diplopia.^8^ Although an eyelid surgery may have sufficed, there is a strong possibility of under/non-correction of the epiblepharon without addressing the orbital component of the disease, the main offender, and also exacerbating the eyelid retraction. When increased fat volume precipitates, an acquired epiblepharon, addressing the primary problem of increased orbital pressure, results in correction of the eyelid abnormality. Performing an eyelid procedure in such a situation will not address the primary pathology and it may exacerbate the eyelid retraction.

In summery we believe that our report is the first to document orbital decompression aimed at treating acquired epiblepharon. A prospective case series of patients with acquired epiblepharon due to increased orbital fat volume, treated with the same technique, is warranted to determine efficacy and side effects.
